# Real-Time Impedance Detection of Intra-Articular Space in a Porcine Model Using a Monopolar Injection Needle

**DOI:** 10.3390/s20164625

**Published:** 2020-08-17

**Authors:** Muhammad Aitzaz Abbasi, Hwijung Kim, Somasekhar R. Chinnadayyala, Ki Deok Park, Sungbo Cho

**Affiliations:** 1 Department of Electronics Engineering, Gachon University, 1342 Seongnamdaero, Sujeong-gu, Seongnam-si, Gyeonggi-do 13120, Korea; aitzazabbasi94@gmail.com (M.A.A.); ssreddy@gachon.ac.kr (S.R.C.); 2 Department of Rehabilitation Medicine, Gachon University, Gil Medical Center Incheon, Incheon 21565, Korea; pmrhwi@gilhospital.com; 3 Gachon Advanced Institute for Health Science & Technology, Gachon University, Incheon 21999, Korea

**Keywords:** electrical impedance monitoring, intra-articular injection, ultrasonography, fluoroscopy, porcine model, monopolar injection needle

## Abstract

Rheumatoid arthritis and osteoarthritis can be treated through specific drug injection into the intra-articular space. Several failures during drug injection attempts with conventional fluoroscopy and ultrasonography in a small area of the intra-articular space have been reported. In this work we present an innovative impedance measurement-based method/algorithm for needle tip positioning to enhance image-guided intra-articular vaccination treatment. A novel algorithm for detecting the intra-articular space in the elbow and knee joints of a live porcine model is reported. An impedance measurement system was developed for biological tissue measurement. The electrical impedance in the intra-articular space was monitored and the needle tip was examined by ultrasonography. The contrast dye was vaccinated and checked using fluoroscopy to confirm that the dye was properly inoculated in the cavity. The electrical impedance was estimated for various needle inclusion profundity levels in saline solution, which were broadly used to evaluate the proposed device for in vivo examinations. Good efficiency was observed in the impedance-based measurements using a monopolar injection needle for intra-articular therapy. To enhance the needle tip positioning for intra-articular therapy, the intended impedance measurement device with a monopolar injection needle can be used as a complement to existing modalities.

## 1. Introduction

Cartilage deterioration is a typical pathological occurrence in different kinds of arthritis, such as osteoarthritis (OA) and rheumatoid arthritis (RA), and is a significant cause of joint dysfunction accompanied by “quality of life” disability in patients [1]. The intra-articular space is characterized by a small spacing between the bone joints. In intra-articular injection therapy, a needle is used to treat sticky capsulitis and joint disorders, along with RA and OA [2,3]. Several imaging modalities have been used to help clinicians determine the factual intra-articular analytic and/or restorative infusion route, with magnetic resonance imaging, computed tomography, fluoroscopy and ultrasound [4]. Depending on the technique, the needles must either be directed to their target position or kept on a straight path to improve the targeting precision and hence quality of care. 

In musculoskeletal disorders, the intra-articular injection technique is a frequently used method, and it is important to align the needle tip accurate with the target for successful treatment. Clinically, several guidance tools are used, such as fluoroscopy and ultrasonography. These methods, though, have several limitations. The ultrasound waves are attenuated as they pass through deep tissue, and it is difficult to accurately discriminate the needle position by using ultrasonography. Fluoroscopy also can be helpful, but the exposure to radiation is unavoidable, and the soft tissue cannot be observed through a fluoroscope. To improve the accuracy, a compensatory method is needed, and we want to use impedance measurement. 

Studies by Tomas et al., indicated that the tilt position of the needle might result in power being transferred to the tip by the tissue, which uproots the needle tip from the objective area [5]. Failure to position the needle accurately can cause misdiagnosis and delayed or ineffective treatment [6,7]. The authors also described the intra-articular treatment of knee OA with ultrasonography-aided needle injection, which led to improved clinical outcomes and reduced treatment costs [4]. In contrast with visionless infusion, ultrasonography and fluoroscopy infusion ensure the improved needle infusion and the area of infusion at the target destinations, thus diminishing infusion-initiated antagonistic results [8]. Ultrasound-guided infusions are quick and radiation-free; concurrently, defective resolution in complex structures makes this methodology less precise [9]. A limitation of this modality is that additional labor is required in order to control the activities of the transducer held with the injection [2]. In the United States, only one of five rheumatologists routinely use musculoskeletal ultrasound, while three of four concur that it should be a typical clinical technique for determination, infusion guidance and treatment comeback assessment. [10]. Fluoroscopy-guided injections involve exposure to radiation, and the representation of delicate tissue anatomy (typically veins and nerves) is restricted with this technique [3,11].

In previous research, algorithms have been designed for needle transportation in 2D space. While these algorithms have improved the needle-focusing accuracy compared with manual needle guidance, some factors, such as physiological development and tissue inhomogeneity, still need to be considered in order to be able to implement these techniques in clinical practice [12]. DiMaio et al., developed a path design and algorithm that connected the needle movement on the exterior of the soft tissue phantom to the tip’s movement in the tissue [13]. Glozman and Shohman, and Neubach and Shoham, solved the forward and reverse needle kinematics for 2D route planning [14,15]. Abayazid et al., developed a 2D ultrasound image-guided steering algorithm [16]. Reed et al., combined a route planner and a stabilizing controller for needle steering on a 2D plane [17]. Force-patterns of displacement have also been studied to enhance the design of interventional needles, microneedles and probes [18,19].

Paul et al., used an echogenic needle for automatic needle steering, but it has no potential in clinical application because of needle breaking due to bending [20]. At some angles, significant reverberation artifacts caused by the highly reflective surfaces of the needles were observed. The needle was not visible from certain angles, as the reflected waves might have scattered away from the transducer [21]. Although the needle could be distinguished, the use of different imaging techniques made it difficult to find the tip of the needle along its axis. Due to the impediments associated with the image-guided infusions detailed above, reciprocal strategies for exact needle positioning and effective medication delivery in obsessive tissues still need to be established.

Sharp et al., showed the feasibility of tissue determination using monopolar and bipolar needle electrodes. In homogeneous volumes, such as biological tissue, monopolar and bipolar electrical impedance setups have very similar operations [22]. Earlier studies showed that the monopolar needle was preferable for intra-articular infusion treatment, as it is simple to use, inexpensive, easily available on the market and no semiconductor technique is required [23,24].

To track the needle’s location accurately in vivo, various studies have investigated tissue characterization with electrical impedance, wherein the electrical properties are determined in real-time using a weak alternating electric current with minimal damage to the tissue subject to the impedance measurements [25,26]. An impedance measurement analyzer with an injectable monopolar needle electrode has been used in various clinical fields for tissue biopsy, peripheral intravenous catheterization and lumbar puncture.

In the medical applications, Kalvøy et al., documented the possibility of tissue impedance (EI) calculations based on the needle’s direction using the finite element method (FEM) reproduction of electric fields [27]. Different tissues can be classified by breaking down a bio-tissue’s electrical properties, determined by its morphological structure and biological condition, such as water substance, fat and sequential tissue [28]. Due to this reason, significant differences are observed in the electrical properties, such as conductivity and permeability, including the impedance in the skin, subcutaneous fat, muscle, and joint cavity. According to Vydyanathan et al., the needle location’s in the human peripheral nerves was identified from extraneural to intraneural compartments by measuring the large difference in electrical impedance [29]. Recently, many studies have determined the electrical impedance of organic cells using electrochemical impedance spectroscopy [30,31]. The EIS-on-a-needle (EoN) technique can calculate the electrical impedance dependent on an electrochemical system, for example, the electrical properties of the sample, and the constant phase element (CPE) on the surface near to the electrodes [26,32]. EoN consists of two main components, a connection part and a detection part. Joho et al., measured pork tissues layers via EoN with respect to entrance profundity [33]. The study reported that the target tissues are located deep inside, and the connection part tends to be in contact with the biological tissues that cause errors at the output. In addition, distortion in the sensor performance can be alleviated by using an insulation layer (SU-8 photoresist) for the contact partition [26]. Kim et al., were the first to introduce an incremental compensation algorithm to boost the detection accuracy [34], and demonstrated the feasibility of the proposed algorithm in distinguishing between normal and cancer tissues ex-vivo [35]. The aforementioned EIS needles suffered from electrode polarization, and semiconductor fabrication was also needed to design electrodes on a needle [35,36]. 

In previous studies, a discrete Fourier transform (DFT)-based impedance analyzer with a monopolar injection needle was developed to track the position of the tip of the needle, and its usefulness was evaluated in porcine tissue. A monopolar needle was used to simultaneously measure the impedance and administer the drug. Electrical impedance monitoring revealed major differences between the dermis, hypodermis, and muscle tissue [23,24].

In this study, a novel algorithm was developed to detect the needle tip in the intra-articular space using EIS with a monopolar injection needle and drug injection in the articular cavity. Here, we demonstrate the ability of the monopolar needle to detect the intra-articular space accurately without any effect on the adjacent tissues. Therefore, the electrical impedance was measured in saline solution at different needle insertion depths to verify that the impedance was not affected by the nearby tissues during monopolar needle injection. Porcine tissue has been chosen in this study because its electrical properties (conductivity, dielectric constant) are identical to those of human skin [37]. Intra-articular injection was administered to porcine knee and elbow joints using the AD5933-based impedance measurement system. An impedance measurement system was developed and the intra-articular space was monitored at 100 kHz. While the needle passed through the skin into the intra-articular cavity, the impedance magnitude of the tissue layers was examined and the location of the needle tip was verified using ultrasonography. Ultrasound was used to protect the blood vessels and sensitive tissues. The contrast dye was injected and examined using fluoroscopy to ensure that the dye was properly injected into the joint space. Based on the available literature and to the best of our knowledge, the present case study is the first report of an impedance measurement system with a monopolar needle for drug injection into the intra-articular space of an animal model. The results demonstrate the effectiveness of the developed algorithm in detecting the intra-articular space using a monopolar needle-based impedance measurement system. 

## 2. Methods

### 2.1. Impedance Measurement System

**Figure 1:**
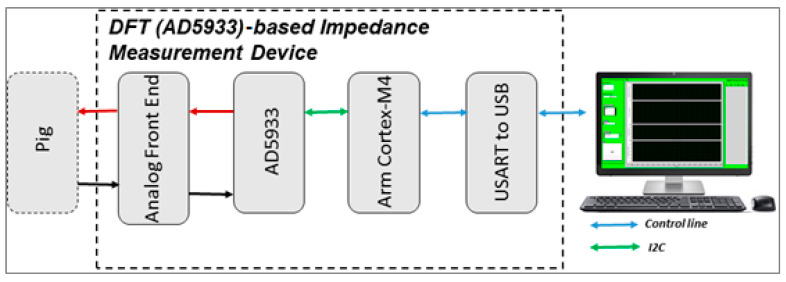
Block diagram of the developed AD5933-based impedance measurement system.

The AD5933 (Analog Devices, Norwood, MA, USA) chip was used for the impedance measurement system. A schematic of the selected AD5933-based impedance analyzer is shown in Figure 1. The AD5933 chip was selected because it is commonly utilized and well recognized for its versatility in selecting the baud rate, as well as its reliability, low cost and low power consumption [38,39]. The AD5933 chip (a) generates an excitation signal only through a voltage-controlled oscillator, (b) has an effective 27-bit direct digital synthesizer (DDS), and (c) achieves impedance by detecting the current passing through the device under test (DUT) and transforming it to voltage using an internal 12-bit, 1MSPS analog to digital converter (ADC) and 1024 point discrete Fourier Transform (DFT) engine to separate the real and imaginary impedance components [40]. The real and imaginary extracted values are stored in a chip register that can be accessed via an I^2^C interface [40]. The ARM Cortex-M4 micro controller was used to control the AD5933 impedance measurement chip through its I^2^C interface. The AD5933 chip requires an external analog front end (AFE) for the accurate measurement of biological samples [38]. The output voltage amplitude vaccinated into the porcine tissue was attenuated by 10×. Therefore, the amplitude was sufficiently small, as per the specifications of the International Electrotechnical Commission for the protection of medical electrical equipment (IEC-60601) [41]. A LabVIEW-based front end was designed for analyzing the results in terms of the magnitude and phase, i.e., the real and imaginary components. The developed framework was operated using LabVIEW with serial communication. The system can measure the impedance continuously at a single frequency and sampling time; it can likewise measure the impedance range at a logarithmic frequency sweep. The precision of the developed impedance measurement device was examined by evaluating the resistor–resistor–capacitor (RRC) circuit, and compared with the measurements of a commercial device (SP-200, Biologic Science Instruments, France). 

### 2.2. Experimental Setup

**Figure 2:**
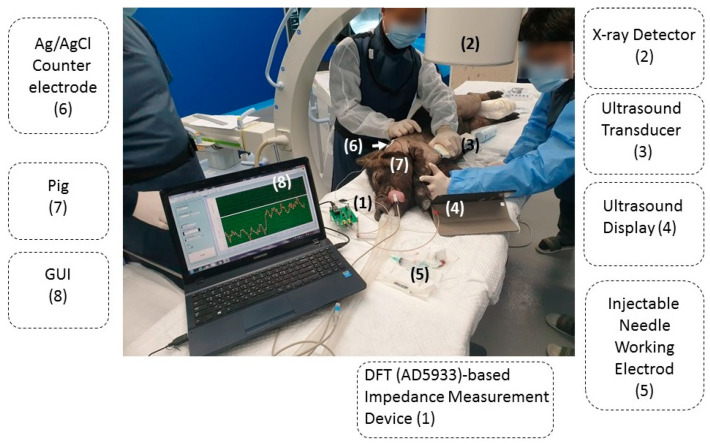
Experimental setup for injecting a drug into the intra-articular space of the experimental animal with an impedance measurement system (1), X-ray detector (2), ultrasound transducer (3), ultrasound display (4), injectable needle working electrode (5), Ag/AgCl counter electrode (6), Pig (7), and LabVIEW GUI (8).

The experimental setup for injecting drugs into the intra-dermal space of a porcine model using a monopolar needle-based impedance analyzer is shown in Figure 2. KNOTUS Animal Care Center took care of the experimental animals. All animal studies were carried out using the protocols of the Gachon University Animal Care Committee. (Animal experiment approval number: KNOTUS IACUC 19-KE-543). For the live porcine animal model, a 7-month-old female pig with a weight of ~32.2 kg was procured from the local market for the animal experimental studies. The room temperature for the animal experiments was adjusted to 22 °C in order to control the influence of temperature on the impedance value. The porcine was administered general anesthesia, using 1:1 v/v% of Zoletil and Rompun, injected to the muscle tissue at 0.1 mL/kg. During the entire experiment, anesthesia was maintained using a respiratory anesthesia machine (isoflurane) to keep the animal sedated for prolonged periods and to prevent any sudden movements. The outer skin surfaces of the knee and elbow joints of the animal were wiped with 70% ethanol and hair was removed using surgical blades. 

The impedance was measured using the AD5933-based impedance analyzer, and the measured value was reported through a LabVIEW-based GUI. A monopolar needle electrode (23 gauge) from Cahlgren Enterprises, Inc., (Gilory, CA, USA) was used as an injectable monopolar needle, and an Ag/AgCl pad was used as the counter and pseudo-reference electrode on the animal skin surface. The injectable monopolar needle working electrode, with a diameter and length of 0.64 mm and 75 mm, respectively, was insulated except for the needle tip. Before administering the injection, the knee joint was scanned using ultrasonography, and the most suitable injection site was selected. An ultrasonic gel (ECO GEL 99, Seung Won Medical Ltd., Incheon, Korea) was used as a supplementary material on the skin of the knees and elbow joints to prevent air gaps among the ultrasonic handheld and skin, so as to acquire a perfect image. The monopolar needle was inserted into the infra-patellar area of both the knees and elbow joints and passed through the skin–subcutaneous muscle layer to enter the intra-articular space. The location of the needle was checked using portable ultrasound equipment (SONON300C, Healcerion Inc., Seoul, Korea). The change in the electrical impedance was monitored in real-time. In one of our previous studies, it was confirmed that the difference in impedance for each tissue layer was statistically significant above 10 kHz. The highest point in the frequency range of the developed system was 100 kHz and the sampling rate was 33 S/s. The electrical impedance spectrum was measured at a single frequency of 100 kHz.

After injecting the needle into the intra-articular space, the electrical impedance was measured while injecting the contrast dye. After dye injection, the joints were examined using fluoroscopy to track/determine whether the contrast agent had been accurately vaccinated into the intra-articular space.

### 2.3. Data Processing

As the joints contain different tissue layers of various thicknesses, and to specifically identify and determine the location of the monopolar needle injection in the intra-articular space, statistical charts known as Shewhart charts were employed.

Let ‘M’ be a set of ‘x’ finite values of the impedance magnitude of the inserted needle in the intra-articular space. These magnitude values are recorded at a constant interval of 30 ms, and can be mathematically expressed as:

(1) $$M=(x_{1},x_{2},\;\ldots ,\;x_{n}{)}^{T}$$

Subsequently, the noise generated during needle insertion effects the impedance magnitude values. Therefore, to remove noise and normalize the magnitude values, a digital low pass Butterworth filter was employed. As the low pass Butterworth filter consists of two parameters, namely the order (*N*) and cutoff frequency ($w_{n}$), it can be mathematically defined as:

(2) $$\left[B,A\right]=Butter\left(N,w_{n}\right).$$

Here, we considered *N* = 1 to boost the measurement system because the time complexity of the measurement system increases with increase in *N*. For $w_{n}$, a hit and trial method was used to determine its value, which, in this case, was determined to be 0.04. The Butterworth function returns two values, namely *B* and *A*, that correspond to the numerator and denominator, respectively. The final normalized value was determined using B and A.

After normalizing the magnitude values, the lower and upper limits of the magnitude of the inserted monopolar needle was identified. For the identification process, the central limit (CL), upper control limit (LCL) and lower control limit (UCL) were computed. In this case, a standard deviation of 3 was selected, which indicates that there is a 99.73% probability that the data points fall between the two limits. The UCL, LCL and CL are defined as:

(3) UCL=μ+(σ×3),

(4) LCL=μ−(σ×3),

(5) CL=μ

where

(6) $$\mu =Mean=D_{1}+D_{2}+\ldots +D_{n}$$

(7) $$\sigma =S.D=\sqrt{\frac{\sum_{i=1}^{Dn}{\left(Di-\bar{D}\right)}^{2}}{N-1}}$$

If the magnitude values are either greater than the UCL or lower than the LCL, then the inserted needle is not current in the intra-articular space. The out-of-range magnitude indicates that the needle is in other tissue layers.

### 2.4. Algorithm for Detecting the Intra-Articular Space

**Figure 3:**
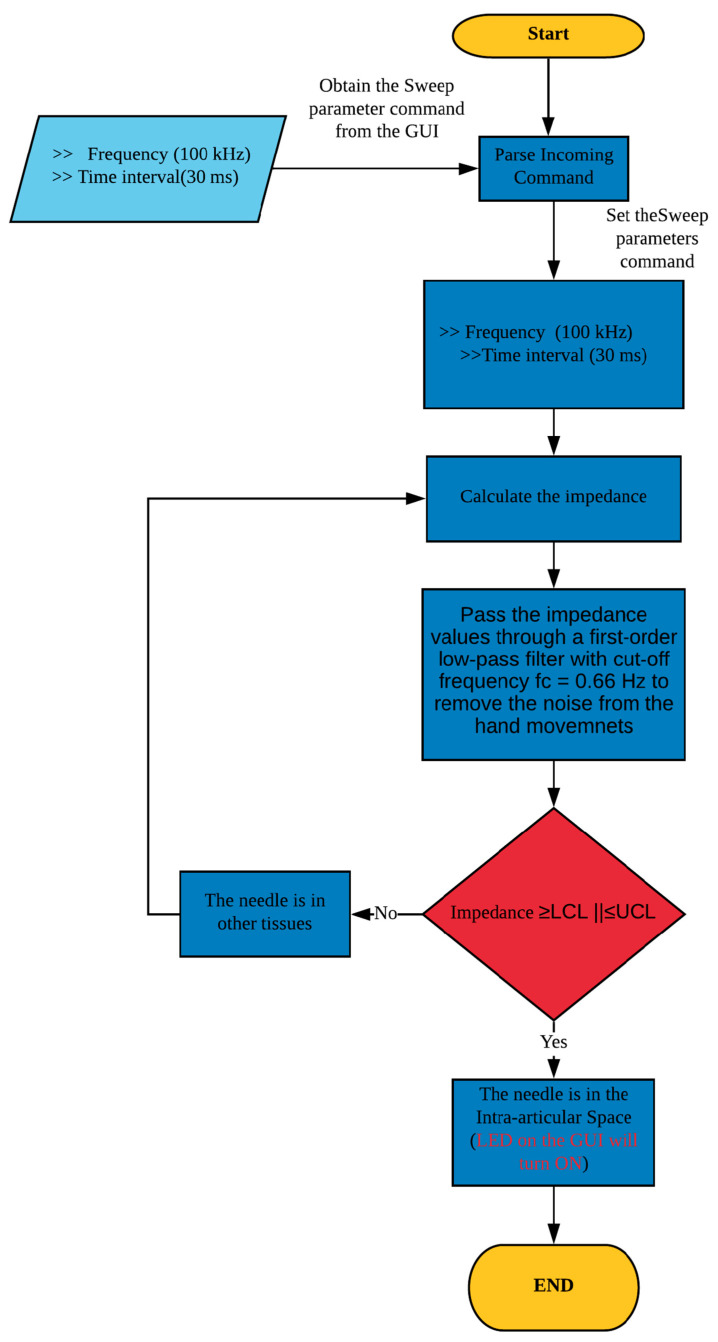
Flow diagram for detecting the needle tip in the intra-articular space.

The entire algorithm process for detecting the monopolar injection needle tip in the intra-articular space is shown in Figure 3. The impedance measurement system first derives the frequency and sampling time from the LabVIEW-based GUI. A maximum sampling time and a sampling frequency of 30ms and 100 kHz, respectively, were used. The system calculates the impedance values, which are then passed through the digital Butterworth first order low pass filter with a cutoff frequency f_c_ = 0.66 Hz. Subsequently, based on the set/prescribed upper and lower control limits, the system decides whether the needle is in the intra-articular space. If the needle is in the intra-articular space, the LED on the LabVIEW-based GUI switches ON and the doctor/operator injects the drug into the cavity. Otherwise, the clinician/operator attempts to locate the cavity position while the system continues to calculate the impedance as per the process/steps mentioned in the flow diagram Figure 3.

## 3. Result and Discussion

### 3.1. Precision Test of The Developed Impedance Analyzer

**Figure 4:**
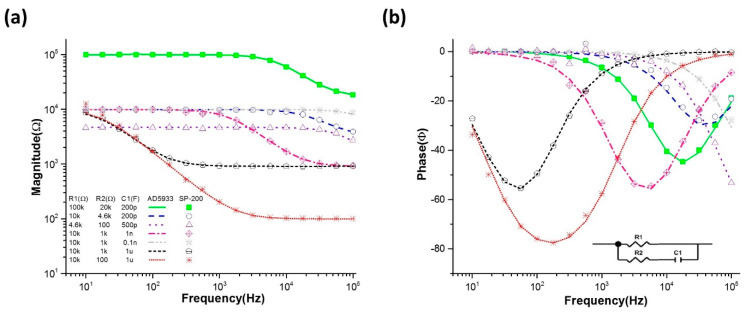
(**a**) Impedance magnitude and (**b**) phase of the resistor–resistor–capacitor (RRC) circuit measured with respect to different C1, R1, or R2 by the AD5933 based impedance measurement system compared with data measured by the commercialized product (SP-200).

The AD5933-based impedance analyzer showed good accuracy in the frequency range of 10 Hz to 100 KHz for impedance magnitudes ranging from 100 Ω to 100 kΩ. Figure 4 shows the magnitude and phase of the impedance spectrum for the RRC circuit measured by the AD5933-based impedance measurement device (line) and commercial device (SP-200, Biologic Science Instruments, Auvergne-Rhone-Alpes, France) for different resistor (1% tolerance) and capacitor values. The RRC circuit shown in the inset of Figure 4b represents the fitted model for different tissue layers [23,42]. The impedance spectra indicate that the developed impedance measurement device based on the AD5933 chip provides good precision at very low cost compared with one of the best impedance analyzers available on the market for biological measurement. 

### 3.2. Saline Solution at Various Concentration Level

To study the effect of the impedance magnitude on the needle insertion depth in the porcine tissue, the electrical impedance measurements were carried out in 0.9% saline solution with varying immersion depths of the monopolar injection needle (2, 4 and 6 cm). The electrical impedance of the saline solution was measured using the fabricated AD5933-based impedance analyzer, with a two-electrode configuration wherein the monopolar injection needle was used as the working electrode, and Ag/AgCl was used as the pseudo-reference electrode/counter electrode in the frequency range of 10 Hz−100 kHz. The fabricated impedance analyzer was connected to a PC via a USB cable to complete the product recreation. The injectable monopolar needle electrode was attached to a height controller stand whose position was movable.

**Figure 5:**
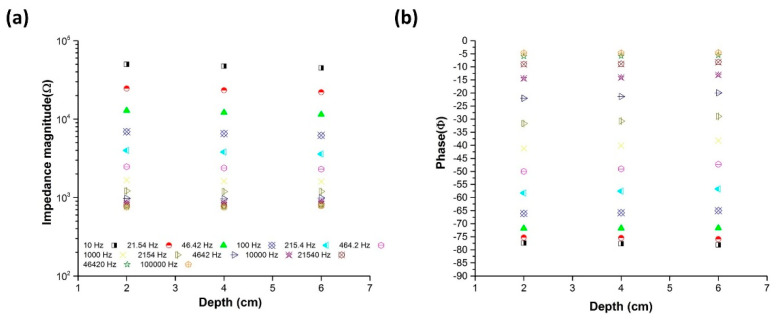
Impedance spectra of the monopolar injection needle measured at different depths in saline: (**a**) magnitude and (**b**) phase.

The current flows out from the tip of the needle electrode due to the insulation layer coated on the needle body. The effective area of the electrode did not vary significantly with the increasing depth of needle insertion. Figure 5 shows the measured impedance spectra of the monopolar injection needle measured at different depths in the 0.9% saline solution, represented in terms of the impedance magnitude (Ω) and phase(φ). At a frequency of 100 kHz, the impedance magnitude did not vary with the depth. Hence, it can be concluded that the monopolar injection needle can be successfully used to locate the needle tip’s position. 

### 3.3. Impedance Monitoring of the Needle Tip in the Intra-Articular Cavity

**Figure 6:**
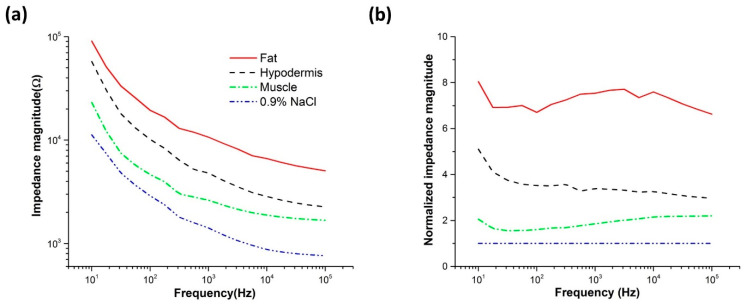
(**a**) Impedance magnitude and (**b**) normalized impedance magnitude spectra at different tissue layers of pork meat at frequencies from 10 Hz to 100 kHz.

For the impedance detection of the intra-articular space, the impedance characteristics of the different tissues (e.g., fat, hypodermis, muscle) were characterized. Figure 6 shows the electrical impedance magnitude measured in the different tissue layer of porcine and the normalized value at frequencies from 10 Hz to 100 kHz. The 0.9% NaCl electrolyte was used instead of the synovial fluid in the intra-articular space, since the conductivity of the synovial fluid is similar to that of the 0.9% NaCl solution (saline) [43]. The impedance magnitude of the muscle tissue layer was lower than the other tissue layers, but higher than that of the 0.9% NaCl electrolyte. The normalized impedance magnitude of the muscle was significantly higher than that of 0.9% NaCl electrolyte at frequencies higher than 10 kHz. Therefore, 100 kHz was selected as the detection frequency for the impedance monitoring of intra-articular space in vivo. 

**Figure 7:**
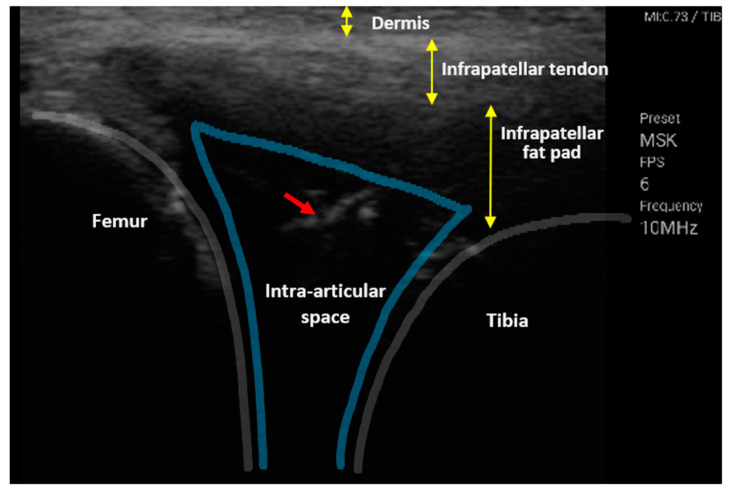
Ultrasonography image that was captured when the needle electrode entered the intra-articular space. The red small arrow shows the needle location and the different depths of the tissue layers indicated by the yellow arrows. The blue boundary shows intra-articular space.

The electrical impedance was monitored at the knee and elbow joints of a live porcine animal using the monopolar needle injection-based impedance analyzer. We concentrated on the real-time impedance monitoring of the monopolar needle injection, tracking the needle penetration from the skin to the intra-articular space, which requires fast signal processing. The impedance was measured at a single frequency with a sampling time of 33 S/s. The fast impedance measurement facilities the real-time determination of the dynamic needle’s location/positioning in different tissue layers. When the monopolar needle passed through the subcutaneous tissue into the joint cavity, the impedance was monitored in real-time, and the change in the needle position was confirmed concurrently using ultrasound, as shown in Figure 7. The red-colored arrow shows that the needle tip was located in the intra-articular space. 

The impedance measured at the knee and elbow joints of the porcine animal is presented in Table 1. The mean and standard deviation of the impedance magnitude data obtained when the needle was in the intra-articular space were calculated. From the mean and standard deviation, the UCL, LCL and CL were calculated using Equations (3)–(5), respectively. There is a significant difference between the impedance magnitudes of the elbow and knee joints; conversely, the knee joints showed a higher conductivity, owing to their excess synovial fluid content compared with the elbow joints. While taking the right knee measurement, the impedance magnitude standard deviation was 5% due to fast animal model breathing and movement artifacts. 

**Figure 8:**
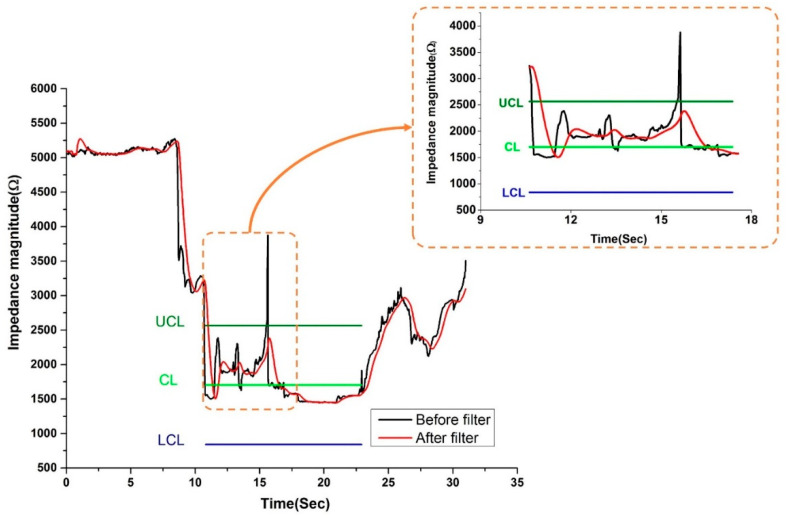
Real-time impedance monitoring at 100 kHz when the monopolar needle moved from the subcutaneous tissue into the intra-articular space.

Figure 8 shows the results of electrical impedance monitoring measured in real-time before and after application of the digital first-order low-pass Butterworth filter. The impedance magnitude changed/varied with changes in the position of the monopolar needle working electrode. The change in the impedance magnitude with the needle position was confirmed by ultrasound, which showed a very high correlation. The steps involved in the injection of the contrast dye into the intra-articular space and the simultaneous measurement of the tissue impedance were as follows: (a) The monopolar needle working electrode was injected into the subcutaneous tissue for a period of 8.85 s. (b) The needle travelled from the subcutaneous tissue to the intra-articular space in 12.12 s (10.77–22.89 s). (c) Loading of the contrast dye into the intra-articular space took 27.98 s. In each step, the changes in the impedance magnitude were measured with the developed AD5933 impedance analyzer, and the needle location was monitored in real-time with ultrasonography.

While the needle was in the sub-cutaneous tissue for 8.85 s, the impedance magnitude was very high. However, when the needle entered the intra-articular space, passing through the sub-cutaneous tissue after a period of 12.12 s (10.77–22.89 s), the impedance magnitude decreased compared with that in the sub-cutaneous tissue. As the loading of the contrast dye into the intra-articular space started at 22.98 s, the impedance again increased slightly. As the dye loading volume increased in the intra-articular space at 27.98 s, the impedance magnitude rose sharply. Each tissue layer has a different moisture content, which makes it unique [28,44]. The electrical impedance was considerably different for each layer, and the position of the needle in a specific tissue could be predicted by monitoring the impedance value. As the fat layer has a relatively low conductivity, the electrical impedance magnitude of fat tissue is higher than that of muscle [23]. In addition, as the joint cavity has a high conductivity due to various fluid contents, the measured impedance here was lower than that of muscle. The electrical impedance increased during the injection because the conductivity of the contrast dye was lower than that of the joint fluid. Through tissue impedance monitoring, the impedance characteristics unique to specific tissue types could be distinguished, with accurate and continuous monitoring of the needle tip. The red line in Figure 8 represents the real-time data obtained after application of the digital first-order low-pass Butterworth filter. The real-time data obtained after low-pass filtering were smoothed out, and the spike at 15 s can be attributed to the artifact generated by the hand movement of the clinician. A first order low-pass filter was utilized to reduce these high-frequency noise spikes.

**Figure 9:**
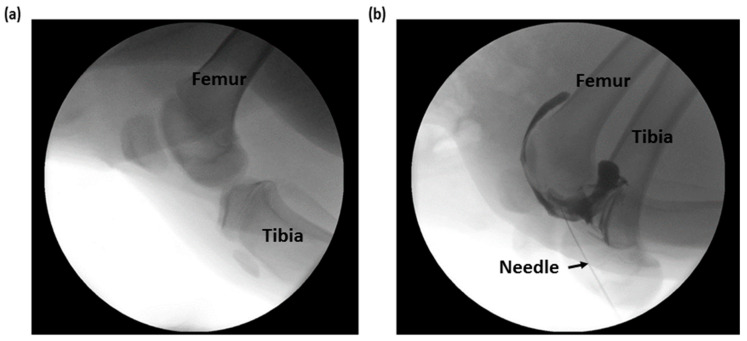
X-ray images by fluoroscopy (**a**) before and (**b**) after injection of contrast in the intra-articular space.

Following injection of the contrast dye, fluoroscopy was performed, and it was confirmed that the contrast dye was successfully injected into the joint cavity. Figure 9a,b shows an X-ray image of the knee joint before and after contrast dye injection. Compared with the knee joint, it was not easy to access the intra-articular space in the elbow, and the classification was limited because there was no significant difference between the impedances measured in the subcutaneous fat and joint cavity.

In the case of the elbow joint of the pig, the difference in the amplitude of the impedance measured in the subcutaneous fat and the articular space was small compared to that of the knee joint, thereby limiting the position of the needle. In the pig’s elbow joint, the needle could not access the articular cavity as easily as it could the knee joint. In future clinical trials, it would be better to focus primarily on the shoulder and knee joints, which are easily accessible. 

There was a difference between the knee joint and the elbow joint, which means that the impedance varies depending on the characteristics of the joint, such as the size of the joint, and the amount of subcutaneous tissue and synovial fluid. In further studies, it will be necessary to collect data by evaluating the characteristics of the impedance for each joint, and study the sensitivity and specificity of the impedance measurement system by increasing population, before evaluating whether it actually works well by applying it in clinical practice.

The conventional anatomical guidance has very low accuracy as compared to the image-guided intra-articular injection therapy [45]. Although the method of image-guided needle injection using ultrasonography and fluoroscopy can be used for intra-articular injection therapy, it has the limitations of image resolution, penetration depth and radiation exposure. Due to the attenuation of the ultrasonic wave in the deep tissues, it is difficult to determine whether the needle tip has reached the intra-articular space. The American Registry for Diagnostic Medical Sonography (ARDMS)’s Registered in Musculoskeletal Sonography (RMSK) certification requires more than 150 clinical experiences in 36 months [46]. Therefore, several attempts have been made to supplement the traditional method using portable and wearable transducers for better diagnosis [47,48]. This study demonstrated the use of an algorithm based on impedance measurement to accurately detect the position of the injection needle tip in the intra-articular space through an animal experiment model. The developed system could be combined with a wearable ultrasound transducer [49] to reduce the labor of the operator and to increase the efficiency of the needle injection therapy in diverse targeted tissues. A comparison of different existing modalities with the proposed method is shown in Table 2.

In this work, we used the conventional injectable monopolar injection electrode, and simply designed the device at a reasonable cost. Given the cost of ultrasound and fluoroscopy equipment, an impedance measurement system can increase the accuracy of injections at low cost. 

Since the location of the needle tip is often ambiguous during intra-articular injection using ultrasound, using a DFT-based impedance measurement system can distinguish the synovial fluid and surrounding tissue and improve the accuracy of injection. Clinically, this is expected to help doctors who have little experience with intra-articular injection, and can be applied in the case of adhesive capsulitis, where the joint space is narrow and difficult to access, and thus increase the success rate of the treatment.

## 4. Conclusions

Needle insertion into soft tissue is a common procedure in intra-articular injection therapy. The feasibility of enhanced intra-articular injection using a DFT-based electrical impedance analyzer with a monopolar injection needle was proposed and tested in a live porcine model. The developed AD5933-based impedance analyzer with a monopolar needle can measure the impedance (100 Ω to 100 kΩ) of biological tissues in the frequency range of 10 Hz to 100 kHz. Dry contrast was vaccinated into the intra-articular cavity of the knee and elbow joints with a monopolar injection, and the impedance was monitored in real-time. Ultrasound was used to protect the sensitive tissues during needle insertion. In addition, the results were validated using fluoroscopy. In conclusion, we demonstrated the potential applicability of a DFT-based impedance measurement analyzer with a monopolar injection needle as a supplement to the image-guided technique for better needle positing in intra-articular injection therapy. 

## Figures and Tables

**Table 1:** Average and standard deviation (STD) of the knee and elbow joints (n = 242).

Type	Right Knee (Ω)	Left Knee (Ω)	Right Elbow (Ω)	Left Elbow (Ω)
Average	1537.3	1367.2	2012.5	2001.8
STD	±88.5	±8.0	±7.4	±12.6

**Table 2:** Comparison of the fabricated impedance-based algorithm’s performance with the existing available methods.

Method	Accuracy	Detection Speed	Price	Penetration Depth Limit	Reference
Conventional anatomical guidance	40%	-	-	-	[45]
Ultrasonography	90%	6 FPS	USD 8340	5 cm	[23,49]
Impedance spectroscopy	99.9%	33 S/s	USD 10	Needle length	This study
